# Sleep Apnea Syndrome in Children: A Retrospective Study of 419 Cases and Polysomnographic Findings with Implications for Rapid Treatment

**DOI:** 10.3390/children12020222

**Published:** 2025-02-12

**Authors:** Lechosław Paweł Chmielik, Anna Kasprzyk, Zuzanna Sala, Tadeusz Chmielik, Grzegorz Jacek Hatliński, Grażyna Mielnik-Niedzielska, Artur Niedzielski

**Affiliations:** 1 Department of Pediatric Otolaryngology, Centre of Postgraduate Medical Education, 01-809 Warsaw, Polandarturniedzielski@wp.pl (A.N.); 2 Department of Pediatric ENT, The Children’s Hospital in Dziekanow Lesny, 05-092 Dziekanów Leśny, Poland; zuzannasala@yahoo.com (Z.S.); hatlinski@infoscan.pl (G.J.H.); 3 Institute of Control and Computation Engineering, Warsaw University of Technology, 00-665 Warsaw, Poland; t.j.chmielik@gmail.com; 4 Department of Paediatric Otolaryngology, Phoniatrics and Audiology, Medical University of Lublin, 20-093 Lublin, Poland; grazyna.niedzielska@wp.pl

**Keywords:** sleep apnea syndrome, polysomnography, sleep study, children

## Abstract

Sleep-related disorders are responsible for many serious health conditions, one of these being sleep apnea syndrome, which arises through various mechanisms. Polysomnography is currently used as the gold standard method for diagnosing patients suffering from sleep breathing disorders, thus enabling accurate and swift clinical diagnosis. **Background/Objectives:** By using the parameters obtained from polysomnography, this retrospective study has aimed to determine the groups of children that are particularly vulnerable to severe forms of breathing disorders, divided according to diagnoses made of tonsillar hypertrophy forms and sleep apnea as well as by the severity grades and types of apnea, to facilitate rapid and appropriate treatment. **Methods:** The subjects were 419 children (from 2017 to 2024) who had been initially diagnosed with sleep apnea syndrome (36.99% girls and 63.01% boys), upon whom various parameters of polysomnography examinations were retrospectively evaluated. The subjects were broken down into groups according to three diagnoses and the types and severities of apnea. The following statistical analyses were used: Kruskal–Wallis tests, post-hoc testing and correlation. **Results:** Tonsillar hyperplasia was found in 334/419 subjects. The total apnea incidence was highest in the total mild severity grouping (53.33%), while the highest total apnea incidence in the apnea type grouping was found in the central apnea grouping at 31.74%, of which the highest sub-group incidence of apnea was found in those with the mild form (25.54%). The highest incidence of apnea was found in its mild form in subjects with pharyngeal and palatine tonsil hypertrophy (19.57%). The highest apnea incidence for those initially diagnosed with sleep apnea was 10.74% for the central apnea type. Children with hypertrophy of the pharyngeal tonsil and palatine tonsils had the more severe form of apnea than in the other cases in terms of AHI. Apnea severity, AHI, ODI and BS below 89% were highest/longest in the peripheral apnea type, whereas the REM sleep time %-age was lowest in this type. Strong positive correlations were found between AHI with ODI and sleep time below 89%, whereas strong negative correlations were found between AHI with average and minimum saturation variables. **Conclusions:** Polysomnography findings of especially mild or severe sleep apnea in children with tonsillar hyperplasia should indicate that surgical treatment is needed as soon as possible.

## 1. Introduction

Breathing/respiration is one of the most fundamental essential processes for life in all living organisms. Appropriate breathing in humans can generally be said to be influenced by the patency of the respiratory tract, while crucially being under the control of the autonomic nervous system, as part of the central nervous system (CNS). There are various mechanisms responsible for breathing disorders that disrupt the process of directing breathing controlled by the nervous system such as central sleep apnea and the patency of the respiratory tract (peripheral apneas), as well as them both acting together. Central sleep apnea is a complete cessation of airflow through the respiratory tract during sleep with a simultaneous cessation of respiratory movements of the chest and abdomen. Such breathing cessation arises from a temporary interruption in the supply of nerve impulses from the brain to the respiratory muscles, there being no obstruction in the upper respiratory tract. The most common cause of central sleep apnea is severe heart failure. This disease can also be associated with damage to the central or peripheral nervous system, (central hypoventilation syndromes, neurological diseases, especially stroke and neuromuscular diseases), along with being at high altitudes, using opioids, any weakening of the respiratory muscles, or disorders in the biochemical regulation of breathing. Peripheral sleep apnea is a disease caused by repeated episodes of breathing cessation (apneas) or a significant shallowing of breathing (hypoventilation) during sleep. This condition occurs despite the correct and sometimes even increased work of the muscles responsible for breathing. Apneas and episodes of hypoventilation are due to the narrowing or closing of the lumen of the airways in the throat, whose airway walls consist only of muscle, there being no cartilage or bone support. Under favorable conditions during sleep, the tension of the muscles that make up the throat wall decreases, thereby narrowing the lumen of the throat, which can lead to its complete closure so that air does not flow into the patient’s lungs. The respiratory muscles are working all the time, trying to overcome the cause of an obstructed throat. Meanwhile, the oxygen content in the body decreases while the concentration of carbon dioxide increases, which activates the autonomic nervous system and increases heart rate, thereby leading to the so-called microawakening. Brain activity then briefly returns to a state resembling wakefulness, but the patient is unaware of waking up. This increases muscle tension and restores the flow of air through the respiratory tract. There is also mixed apnea, which combines the symptoms of central and obstructive sleep apnea and is manifested by a break in snoring and airflow to the lungs and a lack of movement of the chest and abdomen. Another form of both central and obstructive sleep apnea is when the central form appears followed by the obstructive form [1].

**Figure 1:**
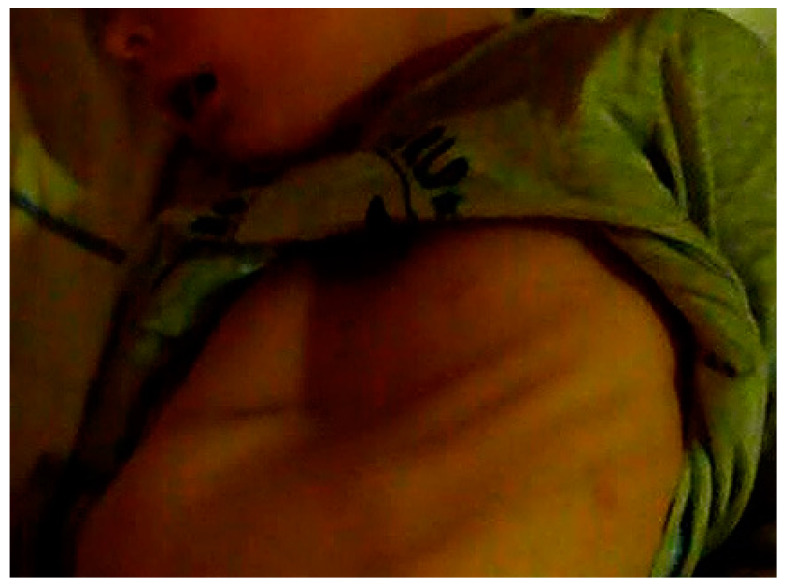
Severe peripheral sleep breathing disorder in children: retraction between the ribs, retraction in the sternum and actuation of additional respiratory muscles.

Breathing disorders can occur in both children and adults during wakefulness or sleep. It should, however, be noted that this disease usually presents itself differently in children when compared to adults (see Figure 1). Disruptions in the breathing pathway can have very serious consequences for the proper functioning and development of the child [2,3,4,5,6]. Breathing disorders during sleep can nevertheless be examined using polysomnography, which measures a number of vital parameters, including an electroencephalogram (EEG), electrocardiogram (ECG), electro-oculogram (EOG), electromyogram (EMG), examining chest and abdominal movements, measuring blood saturation and determining body posture. Polysomnography thereby allows the most important processes that occur in the human body to be recorded during sleep. Based on such means, it is possible to determine, amongst others, such parameters as the AHI (Apnea–Hypopnea Index; average number of such events per hour of sleep), the RDI (Respiratory Disturbance Index; average number of disturbances per hour of sleep, including Apnea–Hypopnea events) and the ODI (Oxygen Desaturation Index; average number of desaturations recorded per hour of sleep) [3,6,7].

The stages of Sleep-Related Breathing Disorder (SRBD) in children can be divided into the following [2,6,7,8,9]:

- Mild form of SRBD; AHI from 1 to 5.
- Moderate form of SRBD; AHI from 5 to 10.
- Severe form of SRBD; AHI above 10.

## 2. Materials and Methods

The study aimed to determine those groups of children that are particularly vulnerable to severe forms of breathing disorders so as to enable rapid diagnosis followed by appropriate treatment.

The study was based on a retrospective analysis of polysomnography investigations that had been performed on children between 2017 and 2024. The inclusion criteria were ages 0-18 years, a history of breathing disorders during sleep, and a diagnosis of chronic upper respiratory tract disease. The exclusion criteria were ages over 18 years, no occurrence of breathing disorders during sleep, nor any diagnosis of acute upper respiratory tract diseases in childhood. The study subjects were 419 child patients who had undergone polysomnography due to suspected sleep apnea. A statistical significance of *p* ≤ 0.05 was adopted throughout and the analyses consisted of comparing three independent group diagnoses consisting of pharyngeal tonsil hypertrophy, sleep apnea and pharyngeal tonsils in conjunction with palatine tonsil hypertrophy. The latter group was jointly constructed because, on their own, there were too few cases of palatine tonsil hypertrophy to be considered as an independent grouping for meaningful statistical analysis. Those patients diagnosed with central or mixed apnea were referred for further neurological diagnostics.

The study was conducted according to the guidelines of the Declaration of Helsinki and approved by the Ethics Committee of the Centre of Postgraduate Medical Education No. 44/2024 of 15 May 2024. To perform statistical analysis, the authors used MATLAB 2023b software.

## 3. Results

The study group consisted of 264 boys (63.01%) and 155 girls (36.99%).

In terms of age, there were no statistically significant differences found. The average age in the study group was 4.99 ± 2.91 years; minimum saturation 76.1% ± 5.6%; average saturation 97.41 ± 1.47%; average AHI Apnea–Hypopnea Index 3.77 ± 12.14 with a maximum of 103.5; and average time below 89% 2.48 ± 20.53 min with a maximum of 385.1 min. Detailed information is presented in Table 1.

The most common form of apnea was mild apnea at n = 223 (53.22%), while the least common was severe apnea at n = 44 (10.5%). The results are presented in Table 2.

The number of cases for each type of apnea found were: absence n = 50 (11.93%), peripheral apnea at n = 116 (27.68%), central apnea at n = 133 (31.74%) and mixed apnea at n = 120 (28.64%) (Table 2).

Table 2 compares the number of cases in the types of apnea to their severity. Apnea was absent in n = 50 (11.93%) cases; the mild form was present in n = 43 (10.26%) children with peripheral apnea; in n = 107 (25.54%) children had central apnea, and in n = 73 (17.42%) had mixed apnea.

The moderate form of apnea was present in 40 (9.55%) cases of peripheral apnea, 22 (5.25%) cases of central apnea and 40 (9.55%) cases of mixed apnea. The severe form of apnea was present in 33 (7.88%) children with peripheral apnea, in four (0.95%) children with central apnea and in seven (1.67%) children with mixed apnea. The results are presented in Table 2.

Child subjects suffering from pharyngeal tonsil hypertrophy showed the following severities of apnea as measured by the number of cases: absence at n = 9 (4.53%), mild apnea n = 84 (20.05%), moderate apnea n = 26 (6.21%) and severe apnea n = 7 (1.67%). Those child subjects with pharyngeal and palatine tonsil hypertrophy demonstrated the following levels of apnea severity: absence n = 13 (3.10%), mild apnea n = 82 (19.57%), moderate apnea n = 58 (13.84%) patients and severe apnea in n = 31 (7.4%). In child subjects with just palatine tonsil hypertrophy, the absence of apneas was found in n = 1 (0.24%), mild apneas in n = 6 (1.43%), moderate apneas in n = 4 (0.95%) and severe apneas in n = 2 (0.48%) patients. The group with developmental defects showed an absence of apneas in n = 1 (0.24%) case and mild apneas in n = 2 (0.48%) instances; however, neither moderate apneas nor severe apneas were found. In children with sleep apnea, the absence of apneas was found in n = 16 (3.82%) cases patients, mild apneas in n = 42 (10.02%), moderate apneas in n = 11 (2.63%), and severe apneas in n = 4 (0.95%) patients. Those children suffering from chronic sinusitis showed just n = 5 (1.19%) cases with mild apneas but none with an absence of apneas nor moderate or severe sleep apneas. In children with nasal septum deviation, n = 2 (0.48%) had mild sleep apneas, and only n = 1 (0.24%) had moderate apneas, but there were no cases of apnea absence nor severe apneas. Finally, children with nasal turbinate hypertrophy showed n = 1 (0.24%) case of moderate apnea, but otherwise no cases of any absent apneas nor mild nor severe apneas. The results are presented in Table 3.

Child subjects suffering from pharyngeal tonsil hypertrophy showed the following types of apneas as measured by the number of cases: absence n = 19 (4.53%), peripheral apnea n = 26 (6.21%), central apnea n = 47 (11.22%) patients and mixed apnea in n = 44 (10.5%). Those child subjects with pharyngeal and palatine tonsil hypertrophy demonstrated the following types of apneas: absence n = 13 (3.10%), peripheral apnea n = 82 (19.57%), central apnea 35 (8.35%) patients and mixed apnea in n = 54 (12.89%). In child subjects with just palatine tonsil hypertrophy, the absence of apneas was found in n = 1 (0.24%), peripheral apneas in n = 3 (0.72%), central apneas in n = 2 (0.48%) and mixed apneas in n = 7 (1.67%) patients. The group with developmental defects showed only n = 1 (0.24%) patient in each of the following groupings: absence of apnea, central apnea and mixed apnea. There no cases found for peripheral apnea. In children with a preliminary diagnoses of sleep apnea, the absence of apneas was found in n = 16 (3.82%) cases, peripheral apneas in n = 3 (0.72%), central apneas in n = 45 (10.74%) and mixed apneas in n = 9 (2.15%) patients. Those children suffering from chronic sinusitis showed no cases of apnea absence nor for the peripheral form, however there were respectively n = 2 (0.48%) and n = 3 (0.72%) cases for central apnea and mixed apnea. In children with nasal septum deviation, there were one case (0.24%) found in each of the following groups: peripheral, central and mixed apnea but there were no cases of apnea being absent. Children with nasal turbinate hypertrophy showed only n = 1 (0.24%) case of peripheral apnea, but otherwise no cases were found of any absent apnea, central or mixed apnea. Finally, children with hypertrophy of the pharyngeal and palatine tonsils and a developmental defect showed no cases in the groups of the absence of apnea, peripheral apnea and central apnea but there was n = 1 (0.24%) case of mixed apnea (see Table 4).

Statistical analyses compared three independent groups (diagnoses) consisting of: pharyngeal tonsil hypertrophy, sleep apnea, and pharyngeal tonsil and palatine tonsil hypertrophy. Other comparisons were omitted because there were too few cases in the remaining diagnoses. For this reason, the diagnosis of palatine tonsil hypertrophy was combined with the group of pharyngeal tonsil cases. The results of these statistical analyses are shown in Table 4.

**Figure 2:**
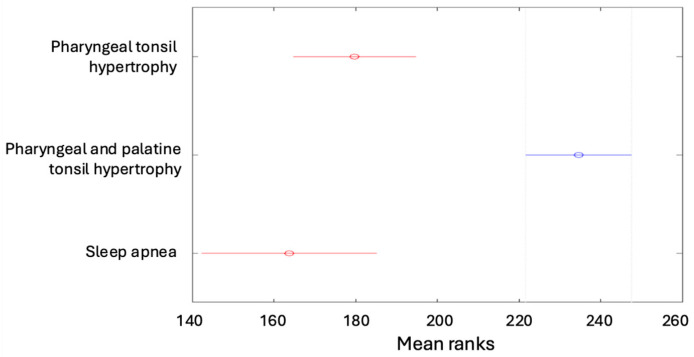
Assessing the severity of apneas according to diagnosis.

**Figure 3:**
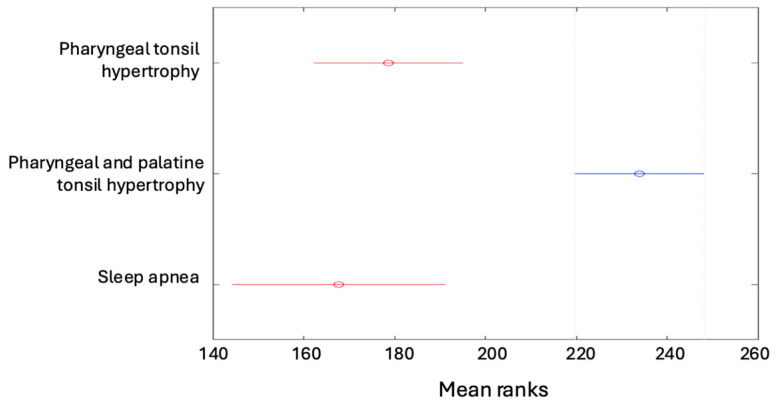
Patients diagnosed with pharyngeal and palatine tonsil hypertrophy had more severe apnea than the other diagnoses.

The Kruskal–Wallis test was performed on the study sample of 406 cases and showed statistically significant differences (*p* = 0.000). Tukey’s multiple comparisons then revealed that children diagnosed with hypertrophy of the pharyngeal tonsil and palatine tonsils had a more severe form of apnea than in other diagnoses. The results are presented in Figure 2. A similar comparison was performed for the dependent variable AHI. The Kruskal–Wallis test showed statistically significant differences (*p* = 0.000). Tukey’s multiple comparisons then demonstrated that children diagnosed with hypertrophy of the pharyngeal tonsil and palatine tonsils had a more severe form of apnea than in the other diagnoses (Figure 3).

**Figure 4:**
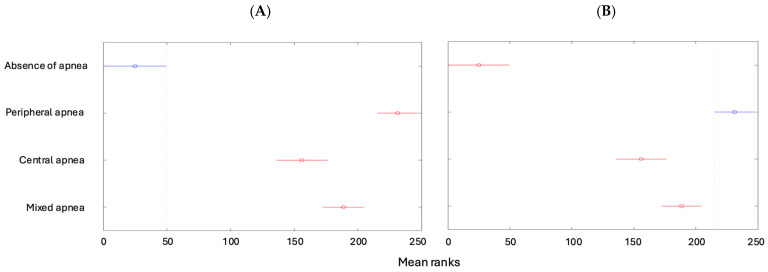
(**A**) Degrees of apnea according to the types of apneas (absence of apnea). (**B**) Degrees of apnea according to the types of apneas (peripheral apnea).

Tukey’s multiple comparisons indicated differences between the mean ranks of the following features: the degree of apnea was lower in children with no apnea than in the other types of apneas (Figure 4A), while it was higher for patients with peripheral apnea than in the other types (Figure 4B).

**Figure 5:**
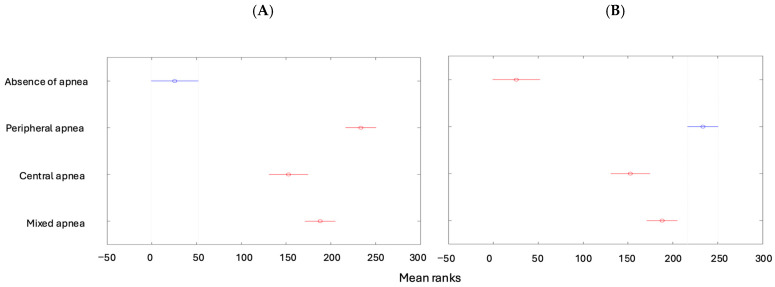
(**A**) The AHI depending on the type of apnea (absence of apnea). (**B**) The AHI depending on the type of apnea (peripheral apnea).

The Apnea–Hypopnea Index (AHI) is lower in children with no apnea than in the other types of apneas (Figure 5A); however, it is higher for patients with peripheral apnea than in the other types (Figure 5B).

**Figure 6:**
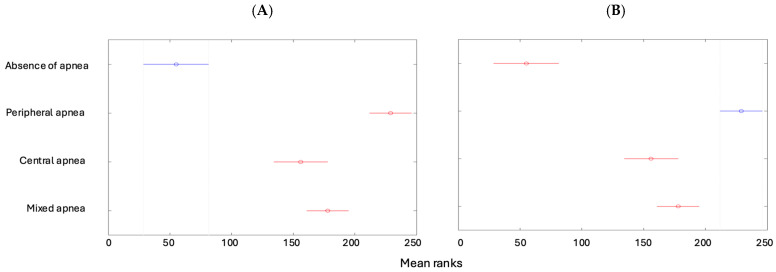
(**A**) The ODI depending on the type of apnea (absence of apnea). (**B**) The ODI depending on the type of apnea (peripheral apnea).

The Oxygen Desaturation Index (ODI) is lower for children with no apnea than in the other types of apneas (Figure 6A), while it is higher for patients with peripheral apnea compared to the other types (Figure 6B).

**Figure 7:**
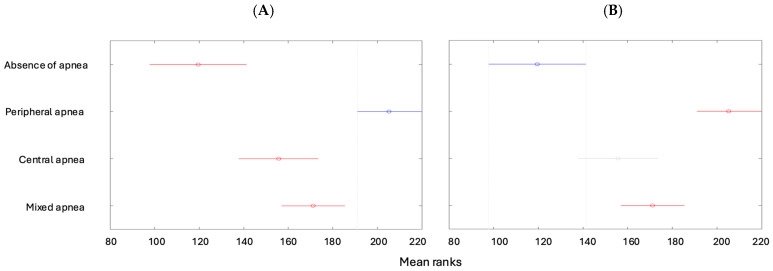
(**A**) The blood saturation time below 89% depending on the type of apnea (peripheral apnea). (**B**) The blood saturation time below 89% depending on the type of apnea (absence of apnea).

The time of blood saturation below 89%, is longer in patients with peripheral apnea compared to the other groups (Figure 7A), while it is shorter in children with no apnea than in the peripheral apnea and mixed apnea groups (Figure 7B).

**Figure 8:**
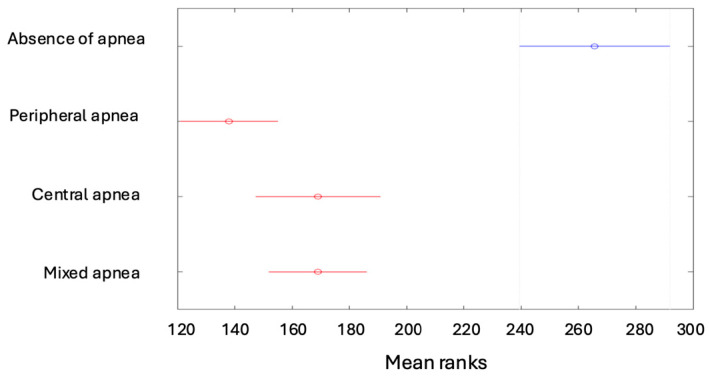
The minimum saturation values depending on the type of apnea.

The minimum saturation revealed higher values for children with no apnea compared to the other groups, while the lowest values of minimum saturation were found in patients with peripheral apnea (Figure 8).

**Figure 9:**
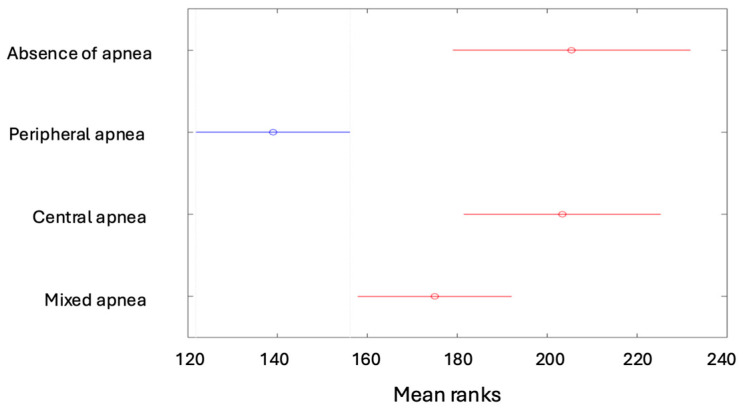
Percentage of sleep time in the REM sleep phase depending on the type of apnea.

**Figure 10:**
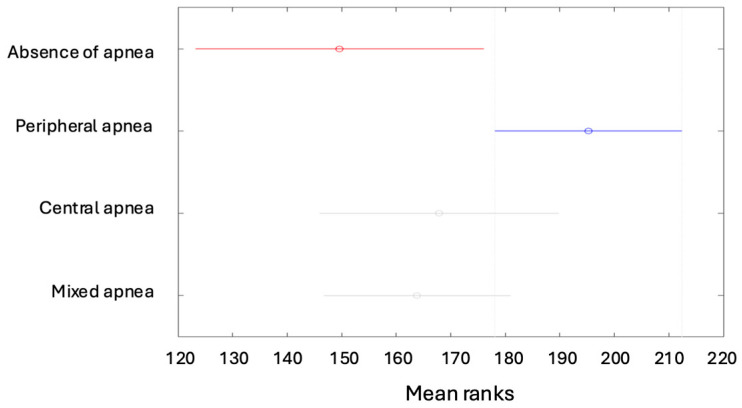
The percentage of time in sleep phase n2 depending on the type of apnea.

The percentage of sleep time in the REM phase was lower in patients with peripheral apnea than in the other apnea type groups (Figure 9). The percentage of sleep time in the n2 phase was longer in patients with peripheral apnea than in those without apnea (Figure 10).

The following statistically significant results were found and are presented in Table 5:

- Very strong positive correlation between the AHI and ODI variables (r = 0.829; *p* = 0.000).
- Strong negative correlation between the AHI and minimum saturation variables (r = −0.594; *p* = 0.000).
- Strong positive correlation between the AHI and time below 89% (r = 0.536; *p* = 0.000).
- Strong negative correlation between the AHI and average saturation variables (r = −0.324; *p* = 0.000).
- Moderate negative correlation between the AHI and rem% variables (r = −0.129; *p* = 0.009).
- This study demonstrated that there were 334 children with tonsillar hyperplasia. In order to determine the scale of this condition, the total number of surgeries performed for tonsillar hyperplasia at our hospital clinic was checked from 2017 to 2024 and found to be 5640. Children with suspected sleep apnea (n = 334) thus constituted 5.92% of this group.

**Table 5:** Correlations found for AHI and other variables (statistically significant results are bolded).

AHI Correlations		
Variable	r	*p*
Age	−0.003	0.957
BMI	0.008	0.939
**Apnea**	**0.911**	**0.000**
**AH**	**0.985**	**0.000**
Snoring (%)	0.040	0.672
**ODI**	**0.829**	**0.000**
**Av. Saturation**	−**0.324**	**0.000**
**Min. Saturation**	−**0.594**	**0.000**
**Time below 89%**	**0.536**	**0.000**
**REM%**	−**0.129**	**0.009**
n1%	0.040	0.421
n2%	0.072	0.149
n3%	−0.074	0.136

## 4. Discussion

The causes of sleep apnea syndrome can be diverse, ranging from acute respiratory infections through to allergies or congenital defects and ending with hypertrophic tonsils [9,10,11,12,13,14,15].

Many studies have emphasized the importance of upper respiratory tract obstruction and its effects on appropriate development and functioning [10,11,16,17,18]. Polysomnography is the basic method used in sleep apnea diagnostics to determine whether sleep apnea exists, what type it is and to what degree it is manifested [4,7,8]. This method is used on children suffering from tonsillar hypertrophy and its use would thus seem justified in identifying patients that need to be diagnosed and possibly treated more quickly than others. Tonsillar hypertrophy is one of the most common conditions in children. The spectra of tonsillar hypertrophy causing a reduction in children’s quality of life is an indication for surgical treatment that will improve mastication in this group of patients [19,20,21,22,23,24]. There were more boys than girls in this study, (63% vs. 37%), which may reflect the beginning of the trend observed in adulthood, where sleep apnea is seen three times more frequently in men compared to women [9,25,26]. Literature findings emphasize that sleep apnea syndrome in children occurs especially when there is hypertrophy of the tonsils [2,14,19,27], which was found in 334/419 subjects of our study initially diagnosed with sleep apnea syndrome. The total apnea incidence was highest in the total mild grade apnea grouping (53.33%) and lowest in the severe grading group, while the highest total apnea incidence in any of the apnea type groupings was found in the central apnea grouping at 31.74%, of which the highest sub-group incidence of apnea was found in those with the mild grade form (25.54%) as opposed to the severe grade, which had the lowest at 0.95%. The highest incidence of apnea was found in its mild grade form in subjects with pharyngeal and palatine tonsil hypertrophy (19.57%) while the lowest was in its severe grade form at 7.4%. The highest apnea incidence for those initially diagnosed with sleep apnea was 10.74% for the central apnea type, whereas the lowest was for peripheral apnea (0.72%). Children with hypertrophy of the pharyngeal tonsil and palatine tonsils had the more severe form of apnea than in the other cases in terms of AHI. Apnea severity grade, AHI, ODI and BS below 89% were highest/longest in the peripheral apnea type, whereas REM sleep time %-age was lowest in this type. Strong positive correlations were found between AHI with ODI and sleep time below 89%, whereas strong negative correlations were between AHI with average and minimum O_2_ saturation variables. The former concordance has been well recognized [28], where indeed it has been proposed that this correlation is sufficiently good for ODI to replace the AHI in screening patients with severe obstructive sleep apnea (OSA), for reasons of lower cost. The latter negative association has also been reported, where it was proposed that lowered average and minimum saturation O_2_ variables can additionally be used with the AHI for treating hypoxia in OSA [29].

The present study has found that children diagnosed with hypertrophy of the pharyngeal tonsil and palatine tonsils had a more severe form of apnea than in other cases, using Tukey’s multiple comparisons following the Kruskal–Wallis test. By examining the numerical distribution of diagnosed cases in the individual types of apneas, it was found that there were no cases of apnea in n = 19 (4.53%) patients with hypertrophy of the pharyngeal tonsil, n = 26 (6.21%) cases of peripheral apnea, n = 47 (11.22%) cases of central apnea in n = 47 (11.22%) patients, and n = 47 (11.22%) cases of mixed apnea. In those children’s groups with pharyngeal and palatine tonsil hypertrophy, the absence of apnea was found in n = 13 (3.1%) cases, peripheral apnea in n = 82 (19.57%) cases, central apnea in n = 35 (8.35%) cases and mixed apnea in n = 54 (12.89%) cases. Children diagnosed with palatine tonsil hypertrophy showed the absence of apnea in n = 1 (0.24%) cases, peripheral apnea in n = 3 (0.72%) cases, central apnea in n = 2 (0.48%) cases and mixed apnea in n = 7 (1.67%) cases, whereas there was only one case found (0.24%) in each of the apnea types —absence of apnea, central apnea and mixed apnea—for those children with developmental defects but no cases were found of peripheral apnea. Among the children with a preliminary diagnosis of sleep apnea, there were no cases of apnea found in n = 16 (3.82%) patients, n = 3 (0.72%) cases of peripheral apnea, n = 45 (10.74%) cases of central apnea and n = 9 (2.15%) cases of mixed apnea. Furthermore, Tukey’s multiple comparisons showed that children diagnosed with hypertrophy of the pharyngeal tonsil and palatine tonsils had a more severe form of apnea than in any of the other diagnoses.

Highly significant and positive correlations were found between AHI and ODI variables (r = 0.829; *p* = 0.000); a strong negative correlation was found between AHI variables and minimum saturation (r = −0.594; *p* = 0.000); a strong positive correlation was found between AHI variables and time below 89% (r = 0.536; *p* = 0.000); and a strong negative correlation was found between AHI variables and minimum saturation (r = −0.324; *p* = 0.000). Moderate negative correlations were found between AHI variables and REM% (r = −0.129; *p* = 0.009). There are many findings regarding obesity and sleep apnea syndrome (SAS); however, our studies showed no significant statistical correlations between AHI and BMI; r = 0.008, *p* = 0.939. In children and adolescents, the standard BMI index has no practical reference in this context, as is the case for adults. This is influenced by two important factors. First of all, the content of adipose tissue is different in boys and girls, and as is already known, BMI takes no account of gender. Another issue is the changes observed in the content of adipose tissue in subsequent years of a child’s life. The nutritional status of children and adolescents is preferably assessed by percentile charts, and this requires further study [30,31,32].

The minimum blood saturation level found of 51.00% will have a significant impact on the functioning of the child’s developing organism, as noted in other studies.

ADHD or epilepsy were not diagnosed in our study group of children. Nevertheless, there are findings regarding the relationship between SAS and epileptic seizures and between anti-epileptic drugs and SAS, as this has been investigated in other studies [23,24,33,34]. We would like to emphasize that all children, in whom central or mixed breathing disorders had been detected by polysomnography, were referred for neurology consultations.

The study subjects did not undergo any endocrinology tests. Nevertheless, we agree that hormonal changes may play an important role in the pathophysiology of SAS in adolescents. This is certainly an important factor to investigate in future studies.

Between 2017 and 2024, 5640 adenotomy and tonsillectomy procedures were performed in the department. Moderate to severe forms of sleep apnea was found to affect 5.92% of children suffering from tonsillar hypertrophy, and the use of polysomnography is thus a good diagnostic method for accelerating surgical treatment as and when required. It should also be noted that children with sleep apnea syndrome can display numerous health disorders ranging from upper respiratory tract development disorders to cardiological conditions [5,6,35,36,37].

The limitation of this study is its retrospective nature in analyzing existing data collected from hospital records; there are no means of obtaining any new information that is not on this database. Further limitations are that stationary polysomnography tests need to be performed in a hospital ward setting, and the screening nature of this study.

## 5. Conclusions

This study confirms the excellent utility of polysomnography as a means of providing rapid and objective diagnoses to be made in children suffering from breathing disorders. It also allows the degree of severity and type of such breathing disorders to be determined. Both factors thereby enable surgical treatment to be rapidly performed whenever deemed necessary. Thanks to polysomnography, the results have pinpointed areas that require appropriate treatment, especially those needing urgent surgical intervention. Outcomes have demonstrated that the mild form of sleep apnea predominates and that sleep apnea syndrome is most common in children with hypertrophy of the tonsils. However, the severe form of sleep apnea is significantly more common in children with hypertrophy of the pharyngeal and palatine tonsils, compared to children with hypertrophy of the tonsils or with other pathologies causing breathing disorders during sleep. Those children with peripheral apnea showed increased AHI but decreased blood saturation and, it also appears, a decreased phase of REM. Surgical treatment was found to be urgently needed for those children exhibiting moderate or severe forms of sleep apnea who had been diagnosed as having tonsillar hypertrophy. Sleep apnea was found to be more common in boys than girls. Further studies are required on sleep apnea syndrome in children to further elucidate all contributing factors (e.g., endocrinology, behavioral disorders, growth and development issues), along with treatment regimens for children suffering from the various forms of breathing disorders during sleep.

## Figures and Tables

**Table 1:** Summary statistics for the study group.

	Min	Max	Median	Average	Standard Deviation	Kurtosis	Skewness
**Age**	1.00	17.00	4.00	4.98	2.91	6.25	1.57
**BMI**	8.90	33.50	15.90	17.34	4.69	5.68	1.65
**AH (index)**	1.00	808.00	28.00	56.22	94.94	27.78	4.48
**Snoring (%)**	0.00	23.60	0.60	2.12	4.32	16.93	3.66
**AHI**	0.20	103.50	3.60	7.27	12.12	26.73	4.35
**ODI**	0.00	92.10	1.20	3.76	9.25	42.29	5.65
**Average saturation**	0.95	98.90	97.80	97.19	4.95	345.88	−17.92
**Minimum saturation**	51.00	98.00	91.00	89.42	5.60	10.91	−2.31
**Time below 89% (min)**	0.00	385.10	0.00	2.47	20.50	295.17	16.15
**rem%**	0.00	25.10	6.70	7.81	4.79	3.30	0.83
**n1%**	3.10	63.50	19.40	19.15	7.25	9.66	1.51
**n2%**	4.40	91.10	56.75	56.54	8.31	8.93	−0.72
**n3%**	0.40	40.60	16.35	16.49	5.26	5.88	0.85

**Table 2:** Breakdown of apnea forms according to severity.

	Absent [1]	Mild Form [2]	Moderate Form [3]	Severe Form [4]	Total
Absence of apnea	50 (11.93%)	0	0	0	50 (11.93%)
Peripheral apnea	0	43 (10.26%)	40 (9.55%)	33 (7.88%)	116 (27.68%)
Central apnea	0	107 (25.54%)	22 (5.25%)	4 (0.95%)	133 (31.74%)
Mixed apnea	0	73 (17.42%)	40 (9.55%)	7 (1.67%)	120 (28.64%)
Total	50 (11.93%)	223 (53.22%)	102 (24.34%)	44 (10.50%)	419 (100%)

**Table 3:** Distribution of cases depending on the diagnosis and severity of sleep apnea in the study group.

	Absence	Mild Form	Moderate Form	Severe Form	Total
Pharyngeal tonsil hypertrophy	19 (4.53%)	19 (4.53%)	26 (6.21%)	7 (1.67%)	136 (32.46%)
Pharyngeal and palatine tonsil hypertrophy	13 (3.10%)	82 (19.57%)	58 (13.84%)	31 (7.4%)	184 (43.91%)
Palatine tonsil hypertrophy	1 (0.24%)	6 (1.43%)	4 (0.95%)	2 (0.48%)	13 (3.10%)
Developmental defects	1 (0.24%)	2 (0.48%)	0	0	3 (0.72%)
Sleep apnea	16 (3.82%)	42 (10.02%)	11 (2.63%)	4 (0.95%)	73 (17.42%)
Chronic sinusitis	0	5 (1.19%)	0	0	5 (1.19%)
Deviated nasal septum	0	2 (0.48%)	1 (0.24%)	0	3 (0.72%)
Nasal turbinate hypertrophy	0	0	1 (0.24%)	0	1 (0.24%)
Pharyngeal and palatine tonsil hypertrophy and developmental defects	0	0	1 (0.24%)	0	1 (0.24%)
Total	50 (11.93%)	223 (53.22%)	102 (24.34%)	44 (10.50%)	419 (100%)

**Table 4:** List of diagnoses made for the apnea patients broken down according to numbers of cases per type of apnea.

	Absence	Peripheral Apnea	Central Apnea	Mixed Apnea	Total
Pharyngeal tonsil hypertrophy	19 (4.53%)	26 (6.21%)	47 (11.22%)	44 (10.5%)	136 (32.46%)
Pharyngeal and palatine tonsil hypertrophy	13 (3.1%)	82 (19.57%)	35 (8.35%)	54 (12.89%)	184 (43.91%)
Palatine tonsil hypertrophy	1 (0.24%)	3 (0.72%)	2 (0.48%)	7 (1.67%)	13 (3.10%)
Developmental defects	1 (0.24%)	0	1 (0.24%)	1 (0.24%)	3 (0.72%)
Sleep apnea	16 (3.82%)	3 (0.72%)	45 (10.74%)	9 (2.15%)	73 (17.42%)
Chronic sinusitis	0	0	2 (0.48%)	3 (0.72%)	5 (1.19%)
Deviated nasal septum	0	1 (0.24%)	1 (0.24%)	1 (0.24%)	3 (0.72%)
Nasal turbinate hypertrophy	0	1 (0.24%)	0	0	1 (0.24%)
Pharyngeal and palatine tonsil hypertrophy and developmental defects	0	0	0	1 (0.24%)	1 (0.24%)
Total	50 (11.93%)	116 (27.68%)	133 (31.74%)	120 (28.64%)	419 (100%)

## Data Availability

The original contributions presented in this study are included in the article. Further inquiries can be directed to the corresponding author.

## Abbreviations

The following abbreviations are used in this manuscript:

AHI	Apnea–Hypopnea Index
CNS	Central Nervous System
EEG	Electroencephalogram
ECG	Electrocardiogram
EOG	Electrooculogram
EMG	Electromyogram
RDI	Respiratory Disturbance Index
ODI	Oxygen Desaturation Index
SRBD	Sleep-related breathing disorder
